# Proximal Co-Translation Facilitates Detection of Weak Protein-Protein Interactions

**DOI:** 10.3390/ijms252011099

**Published:** 2024-10-16

**Authors:** Alina Kordonsky, Matan Gabay, Aurelia Rosinoff, Reut Avishid, Amir Flornetin, Noam Deouell, Taimaa Abd Alkhaleq, Noa Efron, Shoham Milshtein, Julia M. Shifman, Maayan Gal, Gali Prag

**Affiliations:** 1 School of Neurobiology, Biochemistry & Biophysics, The George S. Wise Faculty of Life Sciences, Tel Aviv University, Ramat Aviv, Tel Aviv 69978, Israel; kordonsky@mail.tau.ac.il (A.K.); reut6957@gmail.com (R.A.); amirf@mail.tau.ac.il (A.F.); noamdeouell@mail.tau.ac.il (N.D.); tymaa.abdalkhaleq@gmail.com (T.A.A.); noa.efron@mail.tau.ac.il (N.E.); shoham.milshtein@mail.tau.ac.il (S.M.); 2 Department of Oral Biology, The Goldschleger School of Dental Medicine, Faculty of Medicine, Tel Aviv University, Tel Aviv 69978, Israel; matan.gabay@gmail.com (M.G.); maayaan.gaal@gmail.com (M.G.); 3 Department of Biological Chemistry, The Alexander Silberman Institute of Life Sciences, The Hebrew University of Jerusalem, Jerusalem 9190401, Israel; aurelia.ruimy@mail.huji.ac.il (A.R.); jshifman@mail.huji.ac.il (J.M.S.); 4 Sagol School of Neuroscience, Tel Aviv University, Tel Aviv 69978, Israel

**Keywords:** protein–protein interaction, post-translation modification, ubiquitin receptor, bacterial selection, split chloramphenicol acetyltransferase

## Abstract

Ubiquitin (Ub) signals are recognized and decoded into cellular responses by Ub-receptors, proteins that tether the Ub-binding domain(s) (UBDs) with response elements. Typically, UBDs bind mono-Ub in highly dynamic and weak affinity manners, presenting challenges in identifying and characterizing their binding interfaces. Here, we report the development of a new approach to facilitate the detection of these weak interactions using split-reporter systems where two interacting proteins are proximally co-translated from a single mRNA. This proximity significantly enhances the readout signals of weak protein–protein interactions (PPIs). We harnessed this system to characterize the ultra-weak UBD and ENTH (Epsin N-terminal Homology) and discovered that the yeast Ent1-ENTH domain contains two Ub-binding patches. One is similar to a previously characterized patch on STAM1(signal-transducing adaptor molecule)-VHS (Vps27, Hrs, and STAM), and the other was predicted by AlphaFold. Using a split-CAT selection system that co-translates Ub and ENTH in combination with mutagenesis, we assessed and confirmed the existence of a novel binding patch around residue F53 on ENTH. Co-translation in the split-CAT system provides an effective tool for studying weak PPIs and offers new insights into Ub-receptor interactions.

## 1. Introduction

Protein–protein interactions (PPI) are crucial in all living organisms, influencing various cellular processes [1,2]. The strengths and the dynamics of these interactions are significantly varied depending on their biological roles. Many of the PPIs are dynamic and weak and consequently difficult to study [3,4]. Ubiquitin (Ub) interactions with Ub receptors are usually swift and transient, characterized by high K_on_ (association) and K_off_ (dissociation), consequently characterized by a week or even ultra-week affinities [5,6,7]. Understanding the interactome is an important mission in molecular and cell biology, leading to the development of numerous approaches to studying PPIs, each with its strengths and limitations [8]. Early methods, such as phage display and yeast two-hybrid systems, were among the first to identify and characterize PPIs and are still widely used today [9,10,11]. Recent advances in proximity protein labeling and mass spectrometry technologies have significantly enhanced the discovery of PPIs [12]. However, many eukaryotic PPIs are tightly regulated by transient post-translational modifications (PTMs), making their identification and characterization challenging.

*Escherichia coli* (*E. coli*), which lack most of the eukaryotic PPIs and PTMs, provide an isolated environment that simplifies and facilitates the interpretation of eukaryotic PPI readouts. Previously, we have harnessed a split-dihydrofolate reductase (split-DHFR) and split-chloramphenicol acetyltransferase (split-CAT)-based protein-fragment complementation assay (PCA) to study PPIs and ubiquitylation in *E. coli* [6,13,14]. While most PPI discovery systems efficiently identify strong interactions, many weak interactions, including those between Ub and Ub-receptors, remained elusive.

The coupling of polycistronic transcription and translation processes in bacteria results in the proximity of the nascent polypeptide chains that emerge from polyribosomes. We hypothesized that the co-translation of weak PPI candidates in a polycistronic expression vector would facilitate these interactions, enhancing the readout signal.

In this study, we evaluated the assessment of this hypothesis using several Ub: Ub-receptor systems and other transient PPIs. Using the developed system, we characterized the binding interface of the ultra-weak UBD ENTH (Epsin N-terminal Homology) with Ub.

Epsin (epidermal growth factor receptor pathway substrate 15-interacting protein) functions as a ubiquitin receptor [15,16,17,18]. It plays a pivotal role in clathrin-mediated endocytosis by binding to ubiquitylated cargo and key components of the endocytic machinery [19,20,21,22]. Through its Ub-interacting motifs (UIMs), Epsin facilitates the internalization of plasma membrane proteins. By coupling ubiquitin signaling with the endocytic machinery, Epsin facilitates a concerted sorting and trafficking of ubiquitylated cargo proteins [22].

## 2. Results

### 2.1. Co-Translation Facilitates Readout Signals for PPIs

**Figure 1:**
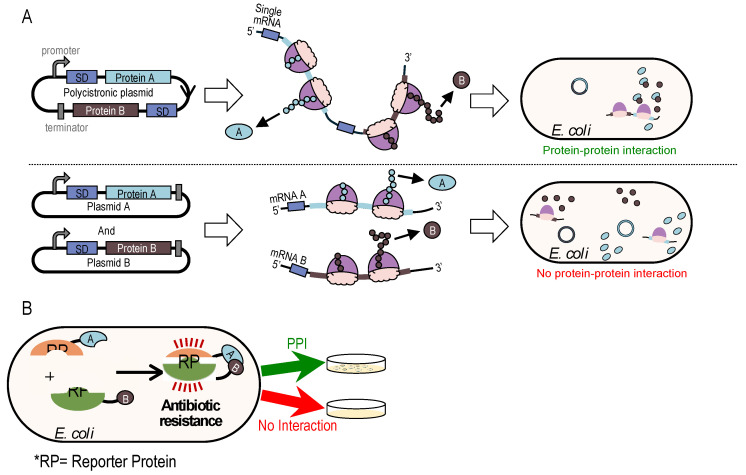
Proximal co-translation from a single mRNA facilitates the detection of PPI. (**A**) In the polycistronic system, two proteins of interest (A and B) are encoded on the same plasmid under the same promoter. The two proteins are co-translated from the same mRNA, bringing them into close proximity and facilitating binding despite even weak affinity. This system minimizes the distance the two proteins need to diffuse to bind to each other. In contrast, when the two proteins are encoded on separate plasmids, each is transcribed and translated independently at different locations within the cell, reducing the likelihood of interaction due to their spatial separation. (**B**) Genetic selection system for testing binding affinity. The folding and activity of reporter proteins depend on the interaction between two proteins of interest (Prey and Bait), each fused to one of the split reporter protein fragments.

In bacteria, translation occurs by polysomes, positioning the nascent polypeptide chains of PPI partners in close proximity when co-translating from the same mRNA. We hypothesized that co-translation of weak PPI candidates would facilitate the molecular search, binding, and formation of protein complexes, thereby enhancing the reporter signal for weak and dynamic PPIs. To examine this hypothesis, we redesigned the split-CAT and split-DHFR reporter systems [12,14] for PPI detection to express both proteins in a polycistronic manner from a single mRNA (Figure 1).

In these systems, a single plasmid expresses both proteins of interest from a single promoter with two constitutive Shine–Dalgarno sequences (SD, a bacterial ribosome binding site) that precede the open reading frames (ORFs) (Figure 1A). This configuration is expected to increase the likelihood of protein complex formation compared to systems where the two proteins are expressed from separate vectors and mRNAs. This is due to the spatial proximity of nascent polypeptides emerging from the same ribosome (Figure 1A).

PPIs facilitate functional assembly of the split-reporters protein, conferring resistance to trimethoprim or chloramphenicol and allowing bacterial growth on selective media. (Figure 1B).

We chose a well-known ultraweak PPI to examine the hypothesis. The EPSIN1-ENTH domains from yeast and zebrafish bind Ub in an ultraweak affinity with *K*_d_ of ~2300 μM [6]. In the newly developed system, the yeast Ent1-ENTH domain (residues 17–152) was fused to the N-terminal fragment of CAT (N-CAT), while the Ub was fused to the C-terminal fragment of CAT (C-CAT), and the two were co-expressed on the same promoter. ENTH: Ub complex formation promotes a functional assembly of the split-CAT reporter, resulting in chloramphenicol resistance and bacterial growth on selective media.

**Figure 2:**
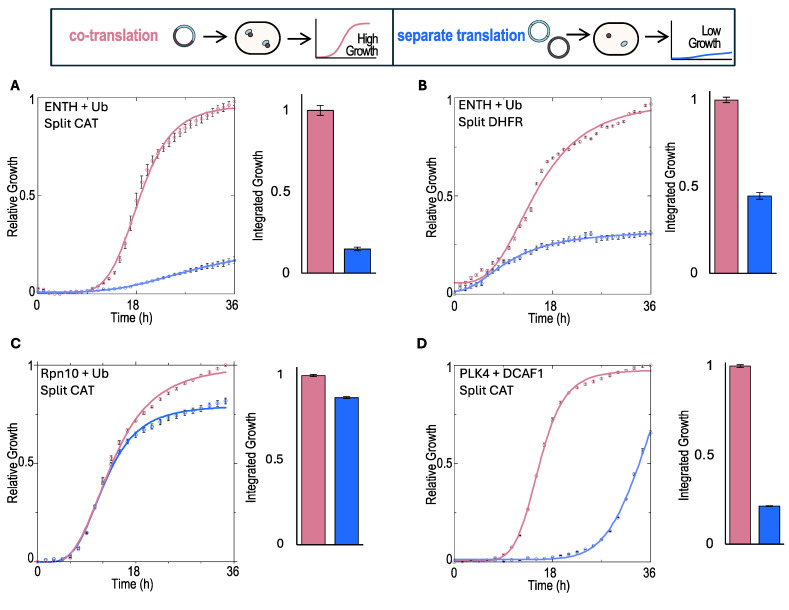
Detection of weak PPIs in proximal co-translation versus separated translation systems. We employed co-translation systems to study weak protein–protein interactions. In all plots, pink represents co-translation systems, and blue represents separated translation systems. (**A**) N-CAT-ENTH and C-CAT-Ub selective growth in 10 μg/mL chloramphenicol media. (**B**) Same as in (**A**), but the split-CAT reporter was replaced with split-DHFR, and growth in minimal selective media was supplemented with 2.5 μg/mL trimethoprim. (**C**) Rpn10 and Ub fused with the split-CAT fragments, showing selective growth in 12 μg/mL chloramphenicol media (**D**) PLK4 and DCAF1 selective growth in 16 μg/mL chloramphenicol media using split-CAT. Quantification of the growth was calculated by the integration of the sigmoidal curves. The relative integral values are presented as bar charts.

As shown in Figure 2A, co-expression from a single mRNA system resulted in a significantly higher growth signal (light pink plot) compared with the expression from two vectors and mRNAs (blue plot). The calculated area under the curve that describes the cumulative growth is presented as a bar plot. These results support our hypothesis that co-translation enhances the interaction between weakly binding proteins.

To test the generality of this phenomenon, we applied the split-DHFR system in a similar manner. As shown in Figure 2B, the split-DHFR system produced results consistent with those obtained using the split-CAT system, with significantly enhanced growth observed in the co-translation system compared to the separated translation system. These results demonstrate the generality of the phenomena independent of the specific reporter.

Next, we evaluated whether this phenomenon is specific to the ENTH: Ub complex or whether the co-translation system possesses a general advantage over other weak PPIs. We tested additional PPIs, including Rpn10:Ub and the recently identified complex of PLK4:DCAF1 [23]. Martin Rechsteiner was the first to identify Rpn10 (also known as S5a) as a Ub-receptor of the proteasome [24]. Rechsteiner also demonstrated two short sequences that bind Ub reside at the C-terminus of S5a. These motifs were further characterized by Emr and Hicke as Ub-interacting motifs (UIMs) [25]. We demonstrated that Rpn10 possesses an additional conserved N-terminal Ub-binding domain (UBD), namely, VWA (Von Willebrand factor type A domain) [26,27]. Rpn10 is well expressed and highly stable in our *E. coli* systems [28]. We expected to find little difference between the binding signals of the separated translation versus the co-translation systems due to the moderate affinity interaction between Rpn10 and Ub. Indeed, smaller yet significant differences were observed in the growth of *E. coli* expressing the two split-CAT fusion systems for the Rpn10:Ub (Figure 2C).

Finally, we asked if the co-translation system can also provide readout for PPIs that are not UB-receptors. We focused on the weak-affinity interaction between Polo Kinase 4 (PLK4) and the Cullin4 substrate receptor (DCAF1), which was recently identified and characterized [23]. We subcloned these two proteins into the two reporter systems (co and separate translation). As shown in Figure 2D, the co-translation system consistently provided significantly higher growth compared to the separated translation system.

Together, these results confirm a general characteristic of the developed system, suggesting that the co-translation of weak PPIs across different protein pairs enhances the readout signal compared with systems where the PPIs are expressed from separate mRNAs.

### 2.2. Homology-Based Model of the ENTH:Ub Interaction 

**Figure 3:**
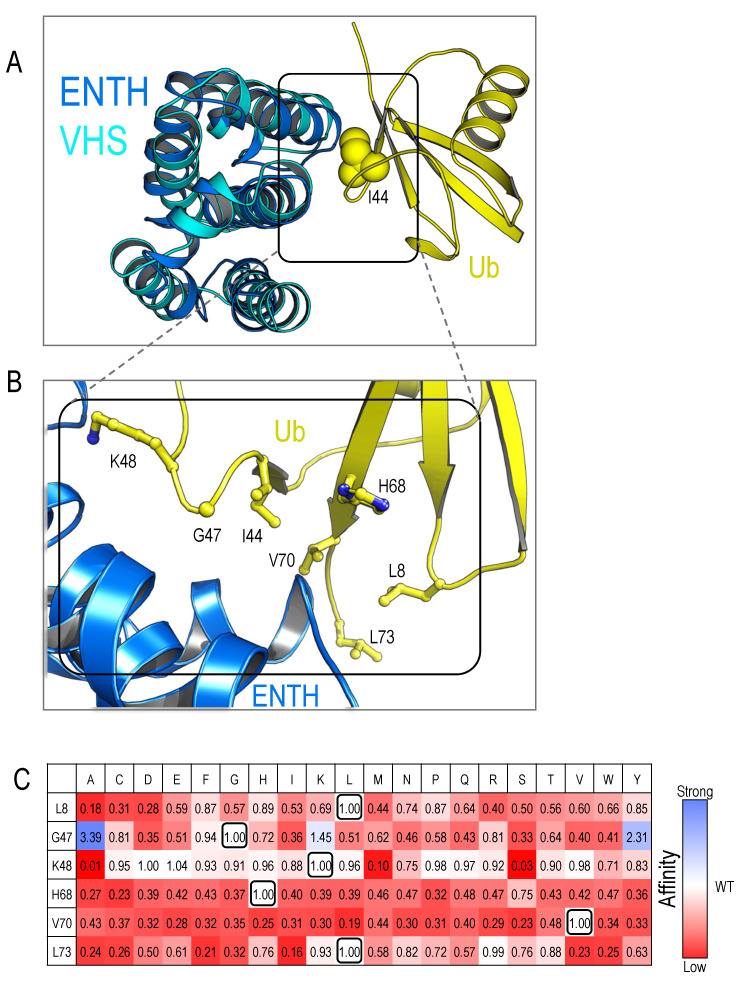
Homology-based structural model of ENTH:Ub. The complex structure of STAM1-VHS:Ub (PDB: 3LDZ) was superimposed on the structure of the yeast Ent1-ENTH domain (PDB: 5LOZ). (**A**) The alignment of the structures of VHS and ENTH suggests the binding patch of Ub on the ENTH surface. (**B**) Zoom-in view of the binding interface, showing the residues on the Ub-I44 patch. (**C**) Heatmap summarizing the relative growth efficiency of the indicated point mutants. The *E. coli* growth assay was performed on selective media with 10 μg/mL chloramphenicol. Growth efficiency compared to the WT Ub, measured by quantification of the spot’s growth over 24 h, integrating the sigmoidal growth curves. Red represents weaker binding, blue represents stronger binding, and the wild-type residues at each position are framed in black.

The reliable readouts of dynamic and weak interactions by the proximal co-translation system encouraged us to further characterize the structural models of ENTH:Ub interaction. We previously determined the crystal structures of yeast and zebrafish Epsin-1 apo ENTH domains [Protein Data Bank (PDB): accession codes: 5LOZ and 5LP0, respectively] [6,29]. These domains share a similar fold with VHS domains [30]. The structure of the VHS domain in a complex with Ub was determined by Hurley and co-workers [30]. They highlighted the importance of tryptophan and leucin residues that interact with the hydrophobic Ile44 patch on Ub. Interestingly, these two key residues are missing in the yeast and the zebrafish ENTH domains, suggesting that their affinity to Ub may be significantly reduced or that binding is mediated via an alternative interface. Our Surface Plasmon Resonance (SPR) measurements confirmed an ultra-weak affinity between ENTH and Ub, with *K*_d_ of approximately 2300 μM [6]. However, due to this low affinity, we could not obtain well-diffractable crystals of the complex. The Parsimony structural model for the complex is based on the evolutionary homology. Using this model, we proposed that ENTH binds Ub through a similar interface as VHS. Structural alignment of the yeast ENTH domain onto the STAM1-VHS domain in the VHS:Ub complex (PDB: 3LDZ [31]) generated a structural model for the ENTH:Ub interaction (Figure 3A). The alignment yielded a root mean square deviation (RMSD) of 3.26 Å over 112 Cα atoms. This model was previously validated by binding experiments involving several point mutants based on structural predictions [6].

Most Ub-receptors recognize the Ub-I44 patch [32,33,34,35,36,37]. To further analyze the binding interface at the Ub side and to develop a general assessment tool for assessing UBDs:Ub interfaces, we constructed a saturated focus library of Ub point mutations in the co-translation PPI reporter system. We selected six residues surrounding the Ub-I44 patch, which we hypothesized to play a key role in interactions with various Ub-receptors (Figure 3B). To investigate how these residues influence binding, we systematically substituted each residue with all 19 alternative amino acids. We then measured the growth efficiency for each of the variants against the wild-type ENTH domain. The relative cumulative growth of the 120 comparative experiments is summarized in the heatmap (Figure 3C).

Detailed data, including the standard deviations for each experiment and an example of the entire data point collected during the experiments of the K48X mutants, can be seen in Supplementary Data Figures S1 and S2. This comprehensive mutational analysis allowed us to assess the impact of each substitution on binding affinity and interaction specificity, providing a detailed understanding of the critical residues that mediate binding to Ub-receptors (ENTH). Our results showed that many mutations in residues L8, K48, H68, V70, and L73 often resulted in growth arrest phenotypes, suggesting their involvement in binding. Intriguingly, substitutions of G47 with alanine (A), lysine (K), or tyrosine (Y) led to increased growth efficiency, suggesting that G47 is a “cold spot” in the interaction. G47 is centered at a β-turn between β3 and β4 of the UBL (ubiquitin-like) β-GRASP domain (a structural motif on ubiquitin involved in protein–protein interactions). The unique structural importance of glycine residues for the formation of β-turns is well known [38]. However, studies by Schulman and Sidhu demonstrated that Ub Variants (UbVs) such as G47R yielded functional proteins with no structural effect on their fold [39]. As seen in their crystal structure of WWP1^HECT^:UbV^P1.1^ (PDB: 5HPS), the RMSD of the wild-type Ub with the UbV is 0.3 Å, which indicates an extremely small difference between the structures and that the overall fold of the protein remains nearly identical. Moreover, the R47 guanidino group forms a new pai (π) interaction with WWP1 I708, and an electrostatic bond with D712 is formed. This new interaction stabilizes the binding between ubiquitin and its partner protein, WWP1 (a HECT (homologous to the E6-AP carboxyl terminus) domain-containing E3 ligase), on their example.

### 2.3. Yeast Ent1-ENTH Possess Two Ub-Binding Interfaces

**Figure 4:**
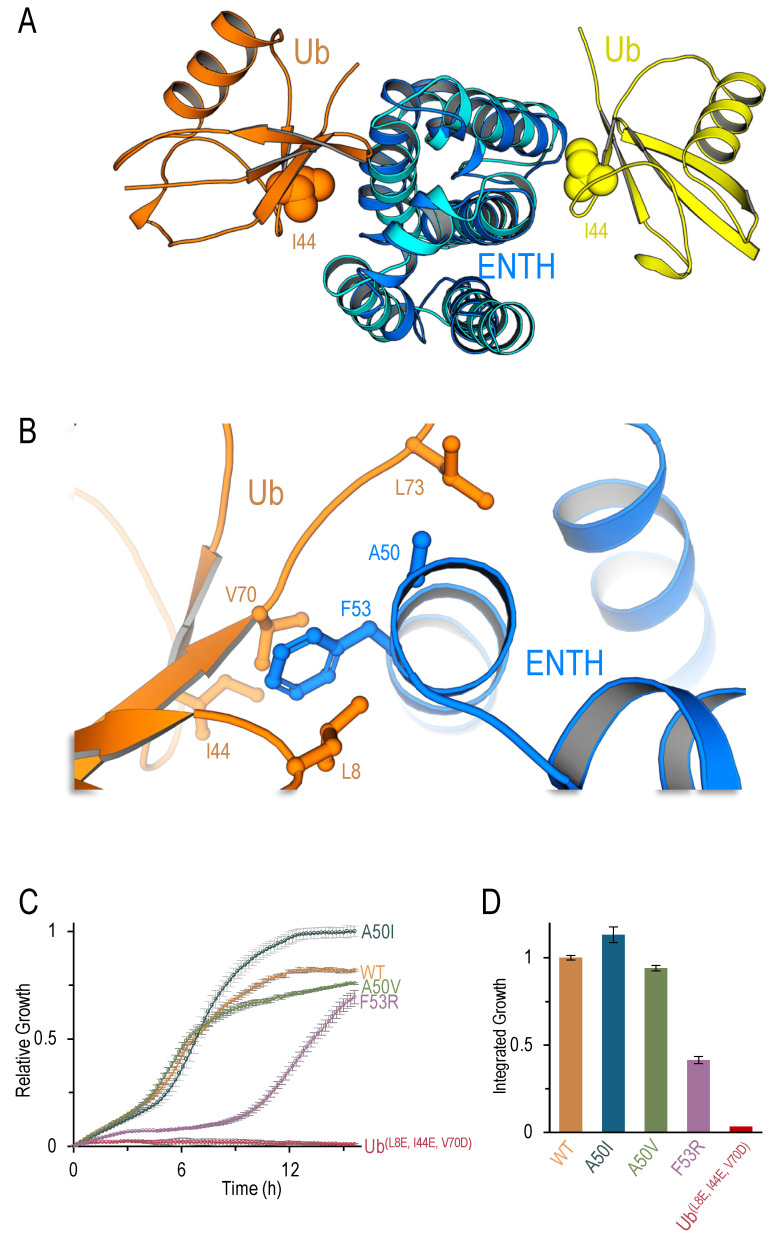
ENTH possesses two Ub-binding patches. (**A**) Overlap of the AlphaFold2 model (Ub in orange, ENTH in blue) with the homology-based model (Ub in yellow, ENTH in light blue). (**B**) Zoom-in view of the ENTH:Ub interaction, highlighting the importance of F53 and A50. (**C**) Growth curves of wild-type ENTH and the indicated mutants in the co-translation system. (**D**) Bar-plot showing the relative cumulative growth based on the integrated growth curves.

Recent advances in structural modeling and the development of AlphaFold2 that can model protein–protein complexes [40] led us to re-examine the modeling of the ENTH:Ub complex. Surprisingly, AlphaFold2 did not replicate the binding mode observed in the VHS:Ub complex presented above, but instead, it predicted models of a new Ub-binding patch located at the other side of the domain compared with that displayed by the homology-based model (Figure 4A). AlphaFold2’s model differs from the homology-based model, likely due to its ability to predict novel interactions based on deep learning rather than evolutionary conservation alone. We performed a careful structural assessment of the suggested models and chose to focus on the rank-1 model generated by AlphaFold2. This model presented the one with the highest predicted local distance difference test (pLDDT) score [41] adopted by AlphaFold to assess the model quality. The model predicted that ENTH residue F53 forms hydrophobic interactions with residues L8, I44, and V70 on Ub (Figure 4B). Moreover, the model also predicted that ENTH residue A50 is positioned near Ub L73 but at a distance of more than 4 Å. To test this prediction, we hypothesized that substituting A50 with a valine or an isoleucine (A50V or A50I), two hydrophobic residues that could potentially increase the interaction with Ub-L73, would increase affinity and enhance growth. Conversely, we predicted that mutation of the key interacting residue F53 to arginine (F53R) would significantly decrease the affinity.

Using the developed co-translation split-CAT tool, we assessed these predictions. As expected, the ENTH F53R mutation exhibited significantly decreased growth compared to wild-type (WT) ENTH (Figure 4C,D). Moreover, the A50I mutation, but not A50V, significantly increased the growth efficiency. This finding corroborates the idea that the space between ENTH A50 and Ub L73 might be filled with bulky groups such as the isoleucine residue. Finally, triple mutation at the Ub-I44 patch (L8E, I44E, V70D) presented a complete growth-arrested phenotype, emphasizing the critical role of this patch in the interaction. These results support the AlphaFold2 model and provide an explanation for the complexity of assessing the effect of mutations on Ub-binding at the Ub-receptor site. The data further suggest that ENTH may possess two distinct Ub-binding interfaces, depending on the context of the interaction.

## 3. Discussion

The physiological relevance and importance of the co-translation assembly of PPIs have recently been highlighted [42]. While many strong and stable PPIs have been discovered and characterized, studies of dynamic and weak PPIs have lagged behind. Our new approach utilizing proximal co-translation of the two interacting proteins offers a new method to facilitate and expedite the identification and characterization of novel PPIs and would enhance our understanding of the proteome. Additionally, our methodology allows us to perform saturation scanning of the binding interface, obtain relative binding affinities for various mutants, and identify key “hot spots” and “cold spots” of the interaction interface [43]. Such scanning is challenging to perform using alternative approaches for ultra-weak affinity PPIs, as demonstrated in this study. 

Our new method takes advantage of the natural bacterial polycistronic operon architecture, where proteins that participate in dynamic or stable complexes are often co-expressed from a single promoter. This setup enhances the likelihood of interaction by bringing the nascent polypeptides into close proximity. Future studies exploring whether proximal co-translation enhances PPI signals in eukaryotic systems could provide new insights into intracellular protein interaction networks. In such systems, it would be advantageous when regulation of the endogenous PPIs is controlled. Although this method offers clear advantages for studying weak PPIs, certain limitations should be considered; for example, in constructing a library of Ub mutations, as we generated in the ENTH:Ub context, sub-cloning is required to introduce the mutants into a different context rather than a simple co-transformation. To address this, we constructed plasmids with KAN^R^ (kanamycin resistance protein) fused to the N-CAT in combination with C-CAT-Ub mutants to facilitate sub-cloning by using reverse selection. 

Another benefit of the system is that, being based on a single plasmid, it offers greater stability and allows interactions to be assessed directly after cloning without the need for plasmid isolation and retransformation into a different bacterial context. This feature significantly streamlines the analysis of new PPI targets and their mutants. Furthermore, the enhanced sensitivity of the co-translational system offers additional advantages for detecting strong PPIs, even those that the separate system is already capable of identifying.

The new discovery that the ENTH domain harbors two Ub-binding patches aligns well with the known role of Epsin in ubiquitylation/clathrin-dependent endocytosis. Downstream to the ENTH domain, Epsin harbors 2 additional Ub-interaction motifs (UIMs), which together enable recognition of short K63 poly-Ub chains and/or membrane cargo marked with multi-mono-Ub moieties. Interestingly, the current structural model does not fit with two Ub moieties linked by K63 ubiquitylation. However, it has been demonstrated that UBDs can intercalate between two Ub moieties in poly-Ub chains or multi-mono-ubiquitylated proteins. For example, Fushman and co-workers demonstrated that the Ub-associated (UBA2) domain of hHR23A has two Ub-binding patches and can simultaneously bind proximal and distal Ub moieties in the K48-ploy-Ub chain [44]. Similarly, some UIMs also possess double faces that interact with the I44 patch of Ub. Stenmark, Wakatsuki, and co-workers demonstrated that the single UIM (double-sided single UIM) of Hrs and some other Ub-receptors bind two Ub moieties to promote endosomal protein sorting [45].

The newly discovered Ub binding patch explains the moderate phenotypes of mutants from the first characterized patch [6]. Given the low affinity and resolution of these complex models, molecular dynamics (MD) simulations and other computational approaches may not accurately depict the two potential ENTH:Ub interfaces. Therefore, future experimental data are essential to fully characterizing these interfaces. To analyze the individual contribution of each Ub-binding patch, one would need to introduce a disruptive mutation in one patch while studying the other. Since both patches are located on the same domain, it is essential to ensure that the mutation in the first patch does not compromise the domain’s stability or the structure of the second patch.

We demonstrated a linear correlation between the affinity of Ub-receptors for Ub as measured by SPR and the relative affinities obtained using the split-CAT system [27]. Consequently, by measuring the affinity between the wild-type Ub-receptor and Ub, it is possible to estimate the contribution of specific residues to binding via point mutagenesis and growth assays in the newly developed split-CAT-based proximal co-translation system. 

The presented approach provides a reliable platform for dissecting the molecular details of weak PPIs, allowing for the precise mapping of interaction interfaces and identification of residues critical for binding.

## 4. Materials and Methods

### 4.1. DNA Cloning and Site-Directed Mutagenesis 

The polycistronic single-operon plasmid was designed based on previous protocols [46,47]. In this plasmid, the ORFs are expressed from a single Ptac promoter, where Shine Dalgarno sequences (ribosome binding sites) precede each of the ORFs as naturally expressed in *E. coli* operons. The first component is the target fused to the N-CAT fragment, and the second component is the C-CAT fused to the second target. If the study is of Ub-receptor and Ub, the UBD is fused to the N-terminus of the N-CAT and Ub is in-frame downstream to the C-CAT. To create the plasmids, the DNA fragments were first amplified by PCR reaction with VeriFi mix (PCRBIO Systems Ltd., London, UK), using primers with overlapping overhangs. DNA fragments were ligated using the Gibson assembly protocol [48]. The ligated plasmids were transformed to *E. coli* Mach1 T1^R^ (ThermoFisher Scientific, Waltham, MA, USA, catalog number: C862003). Plasmids were purified by GeneAid Kit (GeneAid Biotech Ltd., New Taipei City, Taiwan). A list of oligonucleotides used in the study can be found in the Supplementary Data Table S1. The expression of Yeast Ent1-ENTH was previously assessed in *E. coli* [28].

### 4.2. Split-CAT Growth Assay for PPI in E. coli 

A detailed protocol is described in [49]. *E. coli* Mach1 were transformed with either single or double plasmids. Colonies were grown in 5 mL Luria–Bertani (LB) medium for approximately 1–3 h until the cultures reached an OD_600nm_ of 0.3–0.4. The concentration of each culture was adjusted to OD_600nm_ of 0.2 at a final volume of 500 μL. Subsequently, 2.5 μL of the culture was spotted on agar plates containing 8–16 μg/mL chloramphenicol (Sigma-Aldrich, St. Louis, MO, USA) or Davis agar Petri dishes containing 5 μg/mL trimethoprim. Each culture was spotted in triplicate on the same plate, with a uniform distance between spots (Supplementary Figure S1 provides an example plate layout). The plates were covered with a black Petri dish cover and mounted on the scanner in a 37 °C incubator. Spot growth was monitored using SAMPLE (v1.12) (Scanner Acquisition Manager Program for Laboratory Experiments) (https://github.com/PragLab/SAMPLE, accessed on 11 October 2024), a data acquisition software developed in the lab that captures time-lapse scans at defined intervals, typically 60 min. The software and instructions on its use are available in [49]. The split-CAT assays were typically completed within 24–36 h, depending on the interaction affinity and the growth rate of the strain, but faster than the split-DHFR assay, which usually requires longer incubation times for growth to reach measurable levels. To ensure accuracy and reproducibility, each assay was performed three independent times to confirm the reliability of the observed growth patterns. Quantification analyses took place using Fiji (v2.1.0) [50] and KaleidaGraph v5.0.1 of synergy. The assay results were averaged, and the standard deviation was calculated to quantify the variability observed across the replicates. Integrated growth was quantified by calculating the area under the growth curve (integral) for each bacterial culture over the entire time period. This method provides a single value representing overall growth efficiency, allowing direct comparison between the wild-type and mutant strains.

### 4.3. Structural Modeling 

The homology model of the yeast Ent1-ENTH domain (PDB: 5LOZ) was constructed using the human STAM1-VHS domain from the VHS:Ub complex (PDB: 3LDZ) as the template. The ENTH domain was superimposed on the VHS domain using the sequence-independent, structure-based dynamic programming alignment ‘super’ of PyMol (v2.5.4), followed by a few refinement cycles to improve the fit. The model structure was minimized and underwent idealization using Refmac5 of the CCP4 [51]. AlphaFold2 (v2.1.1) [52] modeling was performed using Google Colab and further minimized by Refmac5 (v5.8.0267). All structures were carefully inspected by visualization in PyMol and ChimeraX (v1.3) [53].

## Data Availability

The data presented in this study are available on request from the corresponding author.

## Supplementary Materials

The following supporting information can be downloaded at: https://www.mdpi.com/article/10.3390/ijms252011099/s1.
