# Supplementary material for: Proximal Co-Translation Facilitates Detection of Weak Protein-Protein Interactions
Source: Int J Mol Sci. 2024 Oct 16;25(20):11099. doi: 10.3390/ijms252011099 (PMC11507603; doi:10.3390/ijms252011099)
Supplement: Supplementary file 1 [file ijms-25-11099-s001.zip › ijms-3171176-supplementary.pdf]

Table S1. List of oligonucleotides for cloning and site directed mutagenesis.

No	Name	5' to 3' sequence
1	F_Enth-17_nCAT	CACACAGGAAACAGTATTCATGTCTTCTACCCAGGTCCTGG
2	R_enth152_nCAT	GACGCACCAGCAGCACACCGCTATTCATCCTGTTTCTTCCCTTGATATTC
3	F_Enth-17_cDHFR	GTTCTGGTGCAAGTCAAGGCATGTCTTCTACCCAGGTCCTGG
4	R_enth152_cDHFR	CCTGTTTCTTCCCTTGATATTC
5	F_Rpn10_nCAT	TTCCAGGGGCCCCCGCGGATGGTATTGGAAGCTACAG
6	R_Rpn10_nCAT	GACGCACCAGCAGCACACCGCTATTCATTTTGTCTTGGTGTTGTTC
7	F_PLK4_nCAT	GTTCCAGGGGCCCCCGCGGATGTGGGGTTATCAGAATCGTAC
8	R_PLK4_nCAT	CAGCACACCGCTATTCATACCAGGTTTTCTTCCTATG
9	F_cCAT_Dcaf1	GTATTTTCAGGGCGCCATGGGATCCGCAGAGGATGAGGATGAAGAGGAGG
10	R_cCAT_Dcaf1	GCCTTTGAATTCCAGCCGAACTAGTTCACTCATTCAGAGATAAGATGATG
[a]11	F_Ub-L8X	CGTGAAGACT**NNN**ACTGGTAAGACCATCACC
12	R_ Ub-L8X	GTCTTACCAGT**NNN**AGTCTTCACGAAGATCTG
13	F_ Ub-G47X	GATCTTTGCC**NNN**AAACAGCTGGAAGATG
14	R_ Ub-G47X	CCAGCTGTTT**NNN**GGCAAAGATCAACCTC
15	F_ Ub-K48X	CTTTGCCGG**NNN**ACAGCTGGAAGATGGTC
16	R_ Ub-K48X	CTTTGCCGGA**NNN**CAGCTGGAAGATGGTC
17	F_ Ub-H68X	GTCCACCTTG**NNN**CTGGTACTCCGTCTCA
18	R_ Ub-H68X	GAGTACCAG**NNN**CAAGGTGGACTCTTTC
19	F_ Ub-V70X	CTTGCACCT**NNN**ACTCCGTCTCAGAGGTG
20	R_ Ub-V70X	TGAGACGGAG**NNN**CAGGTGCAAGGTGGA
21	F_ Ub-L73X	GGTACTCCG**NNN**CAGAGGTGGGTGAACT
22	R_ Ub-L73X	GGTACTCCG**NNN**CAGAGGTGGGTGAACT
23	F_Ub	CAGGGCGCCATGGGATCCATGCAGATCTTCG
24	R_Ub	CAGCCGAACTAGTTCACCCACCTCT
25	F_AMP	GCCGCATACACTATTCTCAGAATGAC
26	R_AMP	GGCAGCACTGCATAATTCTCTTACTGTCATGCC
27	F_A50I	AGCTGGCTGAAAAATCATATGATAGCATTTGATTTCTTCGAAATCATGGATATGC
28	R_A50I	GCATATCCATGATTTTCGAAGAATCAATGCTATCATATGATTTTTCAgCC
29	F_A50V	CTGGCTGAAAAATCATATGATAGCGTGATTTCCTTCGAAATCATGGATATGC
30	R_A50V	CCAGCATATCCATGATTTTCGAAGAATCCACGCTATCATATGATTTTTCAGCC
31	F_F53R	GCTGAAAAATCATATGATAGCGCAGATTTCCGCGAATCATGGATATGCTGGAC
32	R_F53R	GTCTTTTGTCCAGCATATCCATGATTTCGCGGAAATCTGCGCTATCATATG

[a] The bold **N** letter in oligonucleotides 11-22 represent the variable nucleotides to create 19 different amino acids other than the wild type at this position. We synthesized all 228 oligonucleotides which are presented by these 12 sequences above.

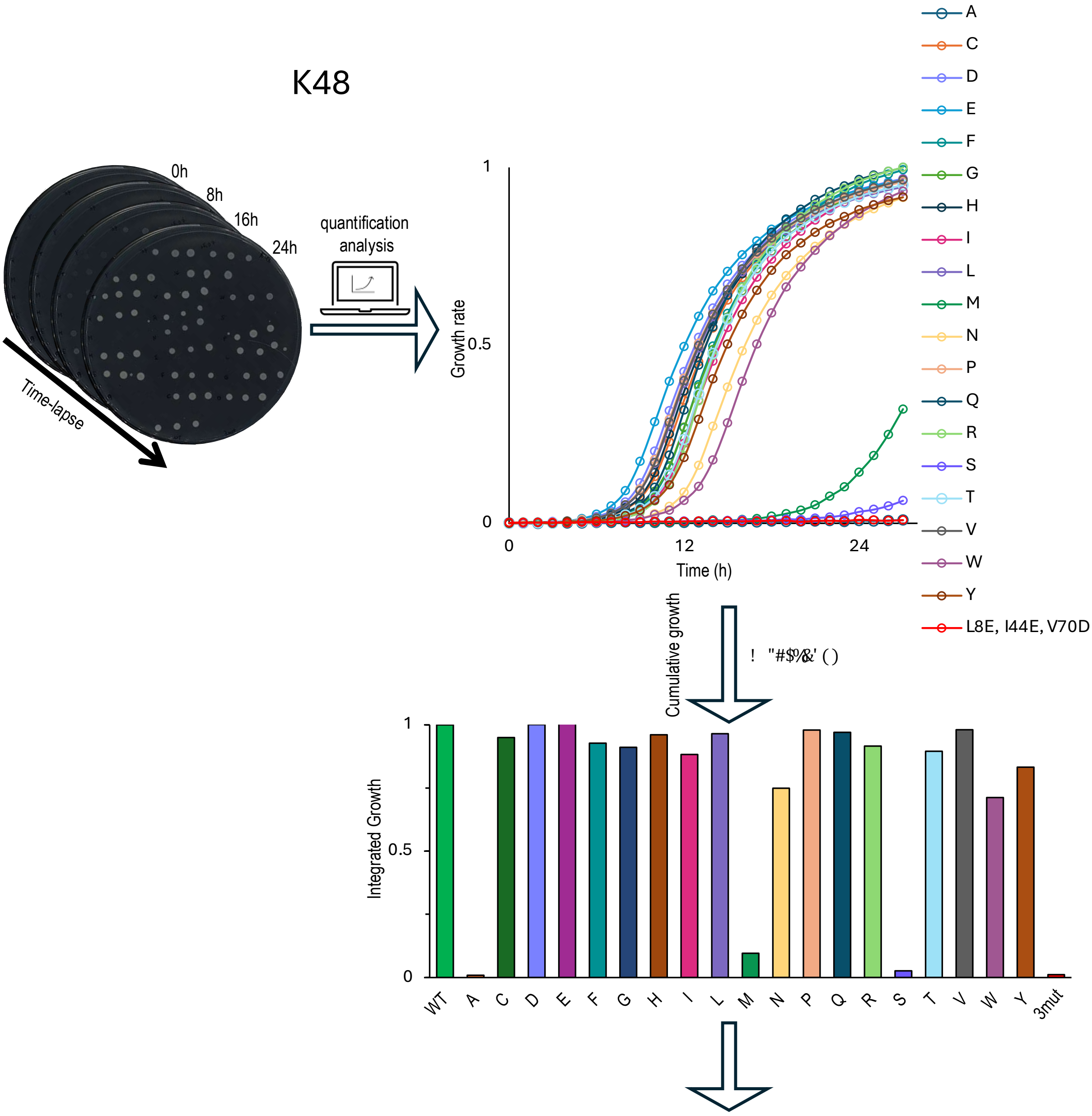

	A	C	D	E	F	G	H	I	K	L	M	N	P	Q	R	S	T	V	W	Y
K48	0.01	0.95	1.00	1.04	0.93	0.91	0.96	0.88	1.00	0.96	0.10	0.75	0.98	0.97	0.92	0.03	0.90	0.98	0.71	0.83

Supplementary Figure S1. *Example of growth data collection from K48X mutants***.** selective agar plate supplemented with 12 μg/mL chloramphenicol was scanned every one hour. Analyses by Fiji and Kaleida Graph yielded the growth curves and the bar plots below for the wild type and each of the 19 mutants. Experiments repeated at least three times to obtain standard deviation as seen in the **Supplementary Figure S2.**

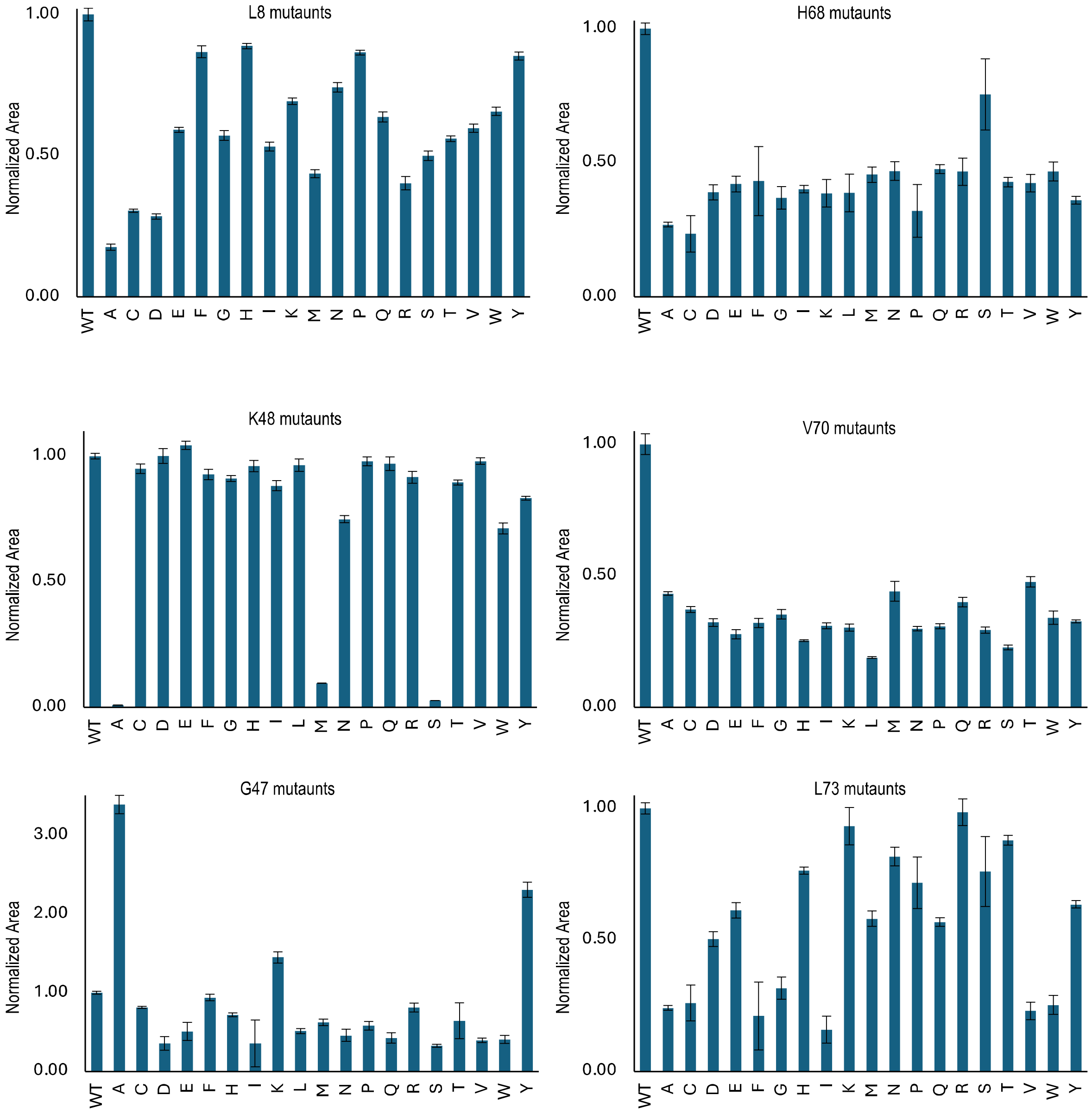

Supplementary Figure S2. *Bar plots showing the relative cumulative growth wild type and mutants.* Wild-type and each of the 19 mutants at each selected indicated mutants is shown with SDs. N=3.
